# Daratumumab Treatment in Severe Anti-NMDAR Encephalitis: Two Case Reports and Literature Review

**DOI:** 10.1212/NXI.0000000000200648

**Published:** 2026-09-09

**Authors:** Nady Ammar, Marjolaine Uginet, Pia De Stefano, Hervé Quintard, Kaveh Samii, Jörg D. Seebach, Josep Dalmau, Patrice H. Lalive

**Affiliations:** 1 Unit of Neuroimmunology, Division of Neurology, Department of Clinical Neurosciences, University Hospital of Geneva, Switzerland;; 2 Neuro-Intensive Care Unit, Department of Intensive Care, University Hospital of Geneva, Switzerland;; 3 Division of Haematology, Department of Oncology, Geneva University Hospitals, Switzerland;; 4 Division of Clinical Immunology and Allergy, Department of Medicine, University Hospitals of Geneva, Switzerland;; 5 Neuroimmunology Program, Institut d’Investigacions Biomèdiques August Pi i Sunyer (IDIBAPS) and CaixaResearch Insitute, Barcelona, Spain;; 6 Service of Neurology, Hospital Clínic de Barcelona, Spain;; 7 Centro de Investigación Biomédica en Red de Enfermedades Raras (CIBERER), Madrid, Spain;; 8 Department of Neurology, University of Pennsylvania, Philadelphia; and; 9 Department of Pathology and Immunology, Faculty of Medicine, University of Geneva, Switzerland.

## Abstract

**Objectives:**

To describe clinical outcomes following daratumumab therapy in severe anti–N-methyl-d-aspartate receptor (NMDAR) encephalitis, including a literature review.

**Methods:**

We report 2 patients with severe anti-NMDAR encephalitis treated with anti-CD38 daratumumab after failure of multiple immunotherapies. Clinical data, treatments, and outcomes were analyzed retrospectively. A PubMed literature review identified 4 additional cases.

**Results:**

Both patients (21 and 19 years) presented with severe anti-NMDAR encephalitis characterized by neuropsychiatric symptoms, seizures, abnormal movements, and dysautonomia. Modified Rankin Scale (mRS) reached rapidly 5 in both cases and required ICU transfer within the first 2 weeks of hospitalization. Despite high-dose methylprednisolone, IV immunoglobulin, plasma exchange, rituximab, and cyclophosphamide, neither patient showed improvement. Daratumumab (1800 mg s/c) was initiated 40 and 14 weeks after onset, respectively. Clinical improvement occurred within weeks after treatment initiation, with gradual recovery of cognitive and functional abilities and ultimately regained functional independence (mRS 0). Literature review identified 4 additional cases of anti-NMDAR encephalitis treated with daratumumab; most patients showed substantial neurologic improvement.

**Discussion:**

These observations suggest that specific plasma cell–targeted therapy may be beneficial in severe anti-NMDAR encephalitis, potentially through reduction of pathogenic autoantibody production. However, current evidence is limited to uncontrolled reports, and further studies are needed to clarify optimal timing, efficacy, and safety.

## Introduction

Anti–N-methyl-d-aspartate receptor (anti-NMDAR) encephalitis is an antibody-mediated disease characterized by a spectrum of neurologic and neuropsychiatric symptoms.^1^ Most patients improve with first-line and second-line immunotherapies, including high-dose corticosteroids, IV immunoglobulin (IVIG), plasma exchange (PLEX), and anti-CD20 (rituximab) or cyclophosphamide.^2^ However, treatment strategies for severe or refractory cases are not well defined. Approximately 20% of patients show insufficient response to second-line therapies (mRS ≥ 4),^3^ and outcomes may remain poor despite escalation to third-line treatments such as proteasome inhibitors or anti-IL6 receptor.^2,4^

In refractory disease, autoreactive B cells may differentiate into long-lived plasma cells that reside in survival niches within bone marrow or inflamed tissues. These cells sustain antibody production and may be resistant to conventional immunotherapies, including anti-CD20 antibodies that deplete B cells and not plasma cells.^5^ Daratumumab, a humanized anti-CD38 monoclonal antibody that depletes plasma cells, is approved for the treatment of multiple myeloma^6^ and has shown efficacy in refractory autoimmune disorders such as systemic lupus erythematosus^7^ and autoimmune hemolytic anemia.^8^

We describe 2 cases of severe anti-NMDAR encephalitis showing marked clinical improvement following daratumumab therapy and review previously reported cases.

## Methods

We performed a literature review using PubMed to identify previously reported cases of anti-NMDAR encephalitis treated with daratumumab. The search was conducted using the following terms: “autoimmune encephalitis,” “anti-NMDA encephalitis,” “anti-NMDAR encephalitis,” and “daratumumab.” Articles were eligible for inclusion if they reported individual patients with anti-NMDAR encephalitis who received daratumumab as part of their treatment regimen and provided clinical information on treatment course and outcome. Reviews, editorials, and articles not reporting individual patient-level data were excluded. In addition to the 2 cases reported here, 4 previously published cases were identified from 2 case reports and 1 case series.

### Patient Consents

Written informed consent was obtained from both patients.

### Case 1

A 21-year-old woman with no previous medical history presented with a first generalized tonic clonic seizure. Neurologic examination, EEG monitoring, and brain MRI were normal. One week later, she returned with neuropsychiatric symptoms, aphasia, abnormal limb movements, brisk reflexes, and dysautonomia. EEG demonstrated diffuse encephalopathy without epileptiform activity. CSF analysis showed pleocytosis (25 cells/µL) with normal protein and glucose levels.

After exclusion of infectious encephalitis, treatment with pulse methylprednisolone (1,000 mg/d) and IVIG for 5 days was initiated, followed by oral prednisone and PLEX. A week later, she required ICU transfer because of impaired consciousness and dysautonomia (modified Rankin Score (mRS) 5). EEG demonstrated extreme delta brush, and CSF anti-NMDAR antibodies were detected on day 14 (cell-based assay flow cytometry, 1:32), confirming the diagnosis. Imaging excluded an underlying teratoma.

Despite treatment with rituximab (2 doses of 1,000 mg, 14 days apart), repeated IVIG, 6 cycles of cyclophosphamide, and 3 cycles of bortezomib, no neurologic improvement was observed. Nine months after onset, the patient exhibited severe cognitive and functional impairment, persistent dysautonomia requiring pacemaker implantation, and complete dependence in daily activities (Functional Independence Measure [FIM] 18; mRS 5) (Figure, A).

**Figure:**
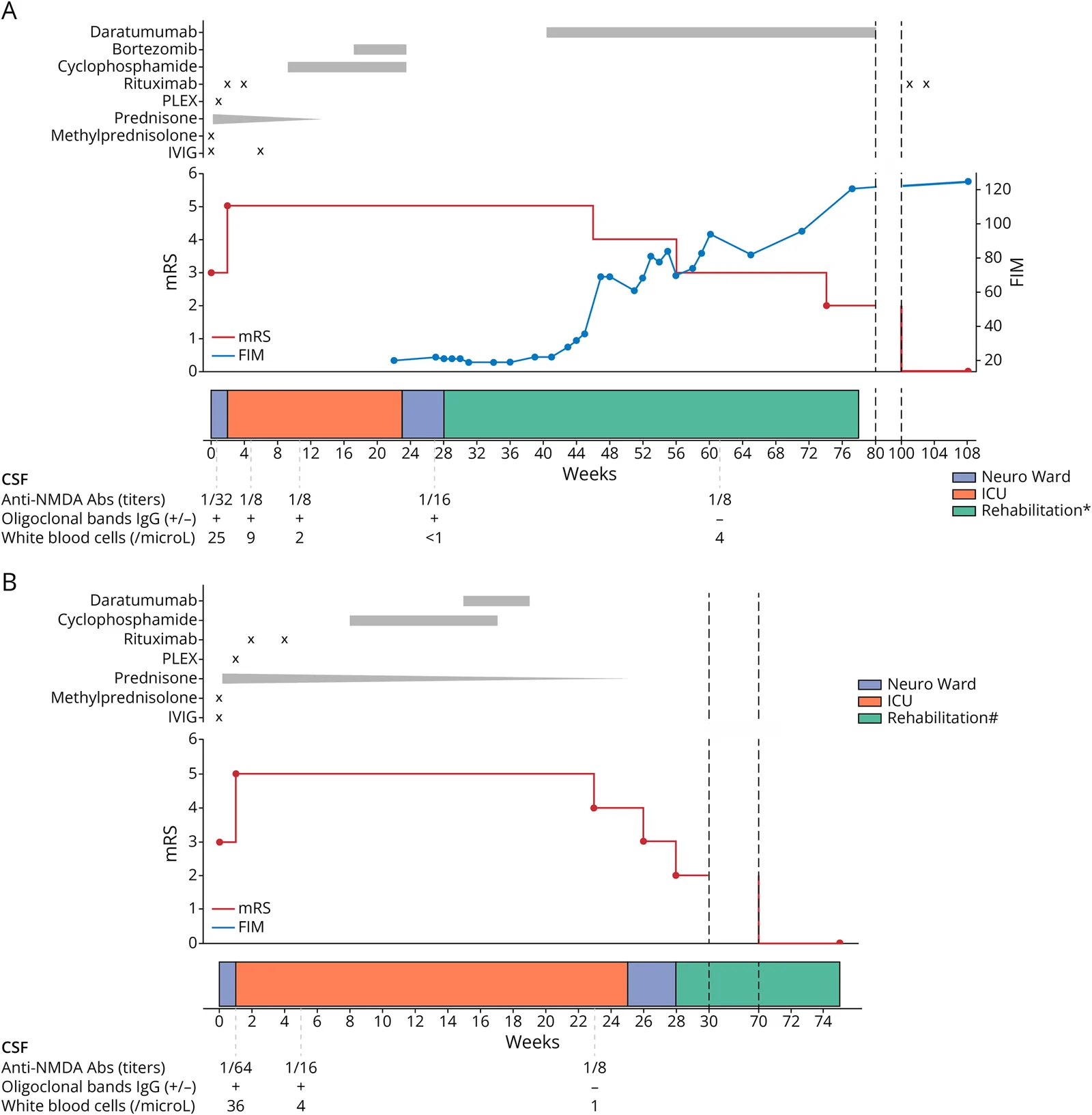
Longitudinal Clinical and Biological Evolution of the 2 Patients Under Immunosuppression Clinical course and severity over time of case 1 (A) and case 2 (B), reflected by the mRS score (case 1 and 2) and FIM score (case 1), as well as illustration of treatment escalation throughout hospitalization. Variation of CSF biological parameter over time: (1) anti-NMDAR Ab titers (live cell-based assay), (2) oligoclonal bands (intrathecal specific IgG synthesis [type 2 or 3]), and (3) white blood cell count (cells/µL). *Rehabilitation in an inpatient setting, allowing FIM measurements; ^#^Rehabilitation in an outpatient setting. FIM = functional independence measure; ICU = intensive care unit; IVIG = IV immunoglobulins; mRS = modified Rankin Scale; PLEX = plasma exchange; WBC = white blood cell.

Daratumumab was initiated 40 weeks after admission using the hematologic protocol (1,800 mg s/c weekly for 6 weeks followed by monthly injections) (Figure, A). Improvement was observed after the third injection (week 3) with constant clinical improvement over the next months. Over the 38 weeks following daratumumab introduction, the FIM increased from 19 to 121 and the mRS improved from 5 to 2. The patient was discharged from hospital 77 weeks after admission. Nine brain MRIs were performed during follow-up, and all were considered normal. At 4-year follow-up, she had resumed her job and other activities without residual symptoms.

### Case 2

A 19-year-old man presented with a 7-day history of progressive visual hallucinations, delusions, aggressive behavior, and somnolence. Neurologic examination revealed orofacial dyskinesia, pyramidal signs, and dysautonomia. CSF analysis revealed pleocytosis (36 cells/µL) with normal protein levels.

Given the high suspicion of autoimmune encephalitis, treatment with pulse methylprednisolone and IVIG was initiated on day 3 for 5 consecutive days. Progressive somnolence, recurrent seizures, and respiratory pauses required ICU admission and mechanical ventilation (mRS 5). EEG demonstrated extreme delta brush, while brain MRI showed isolated leptomeningeal enhancement with no parenchymal lesion. Anti-NMDAR antibodies were detected in both serum (no titer) and CSF (1:64) on day 14, confirming the diagnosis. Tumor screening was negative. Despite high-dose methylprednisolone, IVIG, plasma exchange, rituximab, and subsequent cyclophosphamide therapy, the patient showed no clinical improvement.

Fourteen weeks after clinical onset, daratumumab was introduced together with the fourth cyclophosphamide cycle (Figure, B). Owing to liver insufficiency, possibly related to treatment toxicity, both therapies were discontinued. The patient received 4 infusions of daratumumab in total. The patient rapidly showed gradual neurologic recovery with improvement of cognition and resolution of dysautonomia. Four brain MRIs were performed during follow-up and were considered normal, except for an initial mild bilateral frontotemporal leptomeningeal enhancement attributed to meningitis, which had resolved by week 4 on the second follow-up MRI. He was discharged from the ICU after 25 weeks and regained functional independence by week 26 (mRS 3). At 74-week follow-up, he had resumed his studies without residual symptoms (mRS 0).

## Discussion

Both patients had severe anti-NMDAR encephalitis with no response to second-line immunotherapies. Following initiation of daratumumab, neurologic improvement was observed in both cases, with gradual recovery of cognitive and functional abilities. Both patients ultimately regained functional independence, suggesting a possible association between daratumumab therapy and clinical improvement, although the causal relationship cannot be established. The available evidence stems from uncontrolled case reports and series, making it difficult to separate the effects of daratumumab from delayed responses to prior therapies or spontaneous recovery.

The literature identified only 4 previously reported patients across 3 separate publications (Table).^9-11^ When combined with our cases, all patients were aged 15–21 years and presented with cognitive impairment, abnormal movements, and psychotic symptoms; only 1 had a teratoma.

**Table:** Characteristics of the 2 Reported Patients and Literature Cases of Anti-NMDAR Encephalitis Treated With Daratumumab

	Ammar et al.		Ratuszny et al.^9^	Scheibe et al.^10^	Wang et al.^11^	
Characteristics	Patient 1	Patient 2	Patient 3	Patient 4	Patient 5	Patient 6
Age	21	19	20	16	15	17
Sex	Female	Male	Female	N/A	Female	Female
Symptoms	Psychosis, disturbed consciousness, aphasia, seizure, limb dystonia, severe dysautonomia, tetraparesis	Psychosis, visual hallucinations, aggressivity, seizures, dyskinesias, dysautonomia	Psychosis with hallucinations, disturbed consciousness, personality changes, seizures, orofacial and limb dyskinesia	Psychosis with hallucinations, anxiety and aggressive behavior, status epilepticus, movement disorder	Disturbed consciousness, status epilepticus, orofacial and limb dyskinesia	Psychosis, confusion, cognitive impairment, generalized seizure, limb dyskinesia
Teratoma/cancer	None	None	Teratoma	None	None	None
EEG findings	Extreme delta brush	Extreme delta brush	Extreme delta brush	N/A	Discontinuous epileptic discharges	Extreme delta brush
MRI findings	No parenchymal abnormalities	No parenchymal abnormalities	Bilateral mesial temporal lobe hyperintensities	Diffuse ictal edema	No parenchymal abnormalities	Bilateral frontal lobe hyperintensities
Anti-NMDA Ab CSF (titers)						
At diagnosis	1:32	1:64	N/A	1:400	1:100	1:32
Follow-up	1:16 (week 26)	1: 16 (week 5)	N/A	N/A	N/A	N/A
Last work-up	1:8 (week 60)	1:8 (week 24)	N/A	N/A	N/A	N/A
Anti-NMDAR Ab serum	Positive	Positive	Positive	Positive	Positive	Positive
CSF WBCs at diagnosis (M/L)	25	36	150	16	15	8
CSF oligoclonal bands (onset)	Positive	Positive	Positive	Negative	Negative	Negative
mRS						
Peak	5	5	5	5	5	5
Last follow-up	0	0	3	1	0	1
Residual symptoms at last follow-up	None	None	Independent for basic daily activities but persistent cognitive function deficit	N/A	None	N/A
Immunosuppression received before DARA	Methylprednisolone, prednisone, IVIG, PLEX, rituximab, cyclophosphamide, bortezomib	Methylprednisolone, prednisone, IVIG, PLEX, rituximab, cyclophosphamide	Methylprednisolone, prednisone, IVIG, PLEX, rituximab, cyclophosphamide, bortezomib	Methylprednisolone, prednisone, IVIG, PLEX, rituximab, immunoadsorption	Methylprednisolone, prednisone, IVIG, ofatumumab (in association with DARA)	Methylprednisolone, prednisone, IVIG, ofatumumab (in association with DARA)
Hospitalization time before first DARA treatment (wk)	40	14	28	4	2.5	2.5
DARA dose	1800 mg s/c^a^	1800 mg s/c^a^	16 mg/kg	16 mg/kg	8 mg/kg	8 mg/kg
DARA (cycles)	15	4	10	6	6	5
DARA duration (wk)	60	4	13	5	10	8

Abbreviations: DARA = daratumumab; IVIG = IV immunoglobulins; mRS = modified Rankin Scale; N/A = not available; NMDAR = N-methyl d-aspartate receptor; PLEX = plasma exchange; WBCs = white blood cells.

a Standard hematologic dosing regimen.

All patients received conventional stepwise therapies including high-dose methylprednisolone, IVIG, PLEX, and anti-CD20 before daratumumab initiation. Patients 1, 2, and 3 received additional cyclophosphamide, while patients 1 and 3 also received bortezomib. Wang et al. introduced daratumumab earlier (2.5 weeks after onset) in combination with anti-CD20 antibody (ofatumumab), whereas in other reports, daratumumab was started between 4 and 40 weeks after clinical onset. Other cases used the standard hematologic dosing regimen of daratumumab i.v. (16 mg/kg), whereas Wang et al. used a reduced dose (8 mg/kg). Adverse events potentially related to daratumumab included infections (pneumonia and tracheobronchitis). All patients had severe disability at peak disease (mRS 5). At last follow-up, all patients were either asymptomatic or had only mild symptoms (mRS 0–1) except one that improved but remained moderately disabled (mRS 3) (Table).

The literature review has limitations. The search was limited to PubMed and to published reports, and the small number of identified cases precluded any formal assessment of efficacy or safety. In addition, publication bias and reporting bias are likely because cases with favorable or remarkable outcomes may be more likely to be reported. Therefore, the available evidence should be interpreted cautiously and daratumumab cannot be considered an established treatment for refractory anti-NMDAR encephalitis based on currently available data.

Daratumumab is used in the treatment of multiple myeloma by depleting CD38-expressing plasma cells, including long-lived plasma cells that maintain autoantibody production in refractory autoimmune diseases. Anti-NMDAR encephalitis is associated with a marked intrathecal plasma cell response, with CNS plasma cells/plasmablasts producing anti-GluN1 NMDAR antibodies.^12^ Whether daratumumab directly targets long-lived CNS plasma cells remains uncertain. Its effects may result from limited CNS penetration during neuroinflammation, peripheral depletion of autoreactive plasma cells/B-lineage cells, and modulation of CD38^+^ immune cells. In addition, daratumumab may exert broader immunomodulatory effects through depletion of CD38-expressing NK and T cells.^13^ In our 2 cases, daratumumab was followed by decreased CSF anti-NMDAR antibody titers and resolution of intrathecal IgG synthesis, suggesting a potential benefit of CD38^+^ cell depletion, although delayed effects of previous immunotherapies cannot be excluded.

Although daratumumab was considered well tolerated in our patients, it was discontinued in case 2, along with other drugs, because of hepatocellular failure of undetermined etiology. Daratumumab was not reintroduced because of progressive clinical improvement.

Recent evidence indicates that ICU-dependent refractory anti-NMDAR encephalitis carries a poorer prognosis than immunotherapy-responsive disease,^14^ yet even among patients with a very prolonged (>9 months) vegetative state, approximately two-thirds ultimately achieve substantial recovery. Of interest, third-line immunosuppressive therapies were administered in only 40% of the patients. Hence, the possibility that the recovery observed in our patients was due, at least in part, to a delayed treatment response to second-line immunotherapies cannot be excluded.

Therapeutic strategies beyond first-line and second-line approaches—including rituximab and cyclophosphamide—remain ill-defined, and the efficacy of agents such as tocilizumab or bortezomib is uncertain.^15^ Notably, plasma cell-targeted therapies have been rarely used in the most refractory cases, despite a strong mechanistic rationale, and may represent a valuable option in this setting. Noteworthy, both cases showed a decrease in CSF anti-NMDAR antibody titers, as well as resolution of CSF-specific IgG synthesis, after daratumumab initiation (Figure, A and B). These findings may support the hypothesis that daratumumab can help reduce intrathecal or CNS-compartmentalized anti-NMDAR antibody production. Well-designed prospective studies are now needed to establish the efficacy, optimal timing, and safety of daratumumab in anti-NMDAR encephalitis.
